# Posterior Epidural Migration of a Lumbar Disc Herniation Causing Cauda Equina Syndrome: A Case Report

**DOI:** 10.7759/cureus.2739

**Published:** 2018-06-05

**Authors:** John C Hawkins, Vitaliy P Natkha, Jason Seibly

**Affiliations:** 1 Neurosurgery, Advocate Bromenn Medical Center; 2 DO, Western University of Health Sciences, Lebanon, USA; 3 Neurosurgery, Central Illinois Neuroscience Foundation, Chicago, USA

**Keywords:** posterior epidural migration, disc herniation, cauda equina syndrome, ces

## Abstract

We report an uncommon case of posterior epidural migration of a lumbar disc fragment (PEMLDF) in a patient presenting with acute, progressive back pain, radiculopathy, and weakness. PEMLDF can be mistaken for neoplastic or infectious etiologies on imaging, presenting a diagnostic and management challenge. Our patient underwent an urgent decompressive lumbar laminectomy, which revealed a PEMLDF intraoperatively. He went on to achieve good neurologic recovery.

## Introduction

A herniated lumbar disc is characterized by the displacement of disc material beyond the normal confines of the fibrous annular ring. Due to the presence of the posterior longitudinal ligament, these fragments most often travel in a posterolateral direction [1]. Posterior epidural migration of a lumbar disc fragment (PEMLDF) refers to an unusual trajectory rarely seen with herniated discs, distinguished by dorsal migration around the thecal sac. This can lead to a constellation of symptoms ranging from nerve root irritation and radiculopathy to cauda equina syndrome (CES). Due to this rare and atypical displacement of disc material, they represent a challenge in diagnosis and treatment. We present a case of PEMLDF leading to cauda equina syndrome, and describe the diagnostic and treatment considerations in managing this pathology.

## Case presentation

We present a 40-year-old male with a history of chronic back pain and a recent, acute progression of bilateral lower extremity paresthesias and weakness. Over a seven-day period prior to presentation, the patient experienced a sudden onset of bilateral leg numbness, with no history of trauma or another precipitating event. The right leg was affected more severely than the left, traveling primarily in a distribution down the lateral leg into the dorsum of the foot and great toe. He eventually began having a shooting pain in this same distribution. He also reported a progressive weakness with bilateral ankle and toe dorsiflexion and five days duration of perineal numbness and mild urinary retention. On exam, he was unable to dorsiflex his ankles or great toes against gravity and had reduced sensation in the lateral aspect of both lower legs and the dorsum of bilateral feet. The patient’s Achilles reflexes were also diminished bilaterally, though reflexes were present and brisk at the knee. It is also pertinent that no upper motor neuron signs were present.

**Figure 1:**
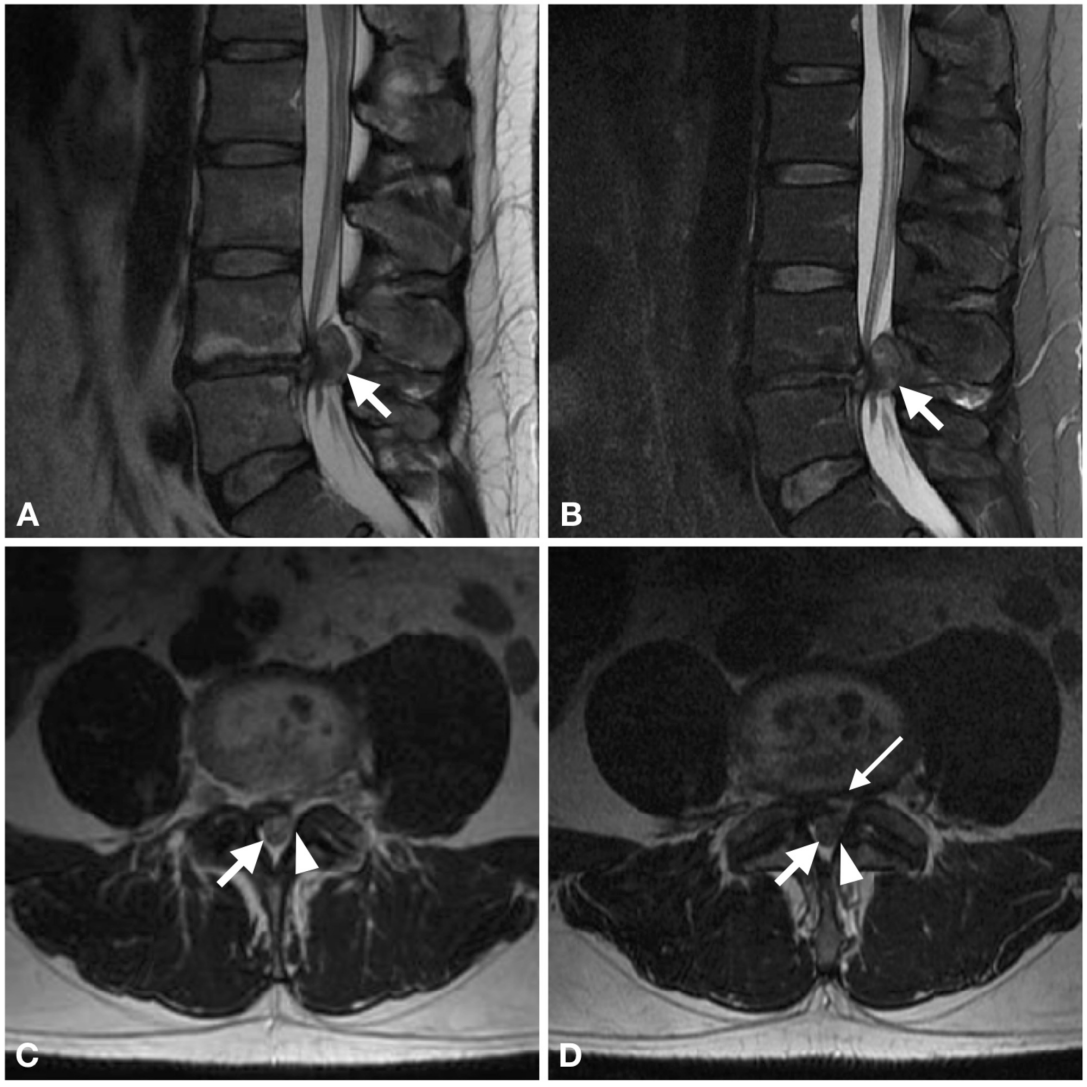
MRI of a 40-year-old male with acute onset of progressive bilateral leg weakness, back pain, and urinary retention Sagittal T2-weighted (A) and T2-weighted with fat suppression (B) images demonstrating a large T2-hyperintense mass (white arrow, A-D) in the L4-5 epidural space. Axial T2-weighted images at the level of the L4 vertebral body (C) and L4-5 disc (D) show the mass present in the left lateral and dorsal epidural spaces, displacing the thecal sac anteriorly and to the right. The mass abuts the left L4-5 ligamentum flavum (arrowhead, C-D), but does not create an obvious connection with the facet joint. It is, however, contiguous with the dorsal aspect of the disc space (skinny arrow, D). MRI: magnetic resonance imaging

Magnetic resonance imaging (MRI) of the lumbar spine revealed a somewhat heterogeneous, but predominately T2-weighted, hyperintense mass in the left lateral and dorsal epidural spaces (Figure 1). There was significant lumbar stenosis present at the level of L4-5 secondary to the mass, with the rightward displacement of the thecal sac. There was no clear connection to the adjacent facet joint although the dorsal mass did seem to be contiguous with the dorsal aspect of the L4-5 disc (Figure 1D).

Due to the acute onset of symptoms, as well as the severity of neurologic involvement, the patient was taken to surgery for exploration and removal of the epidural mass. An L4-5 laminectomy was performed, which revealed a very large dorsally migrated disc fragment that erupted as soon as the ligamentum flavum was removed. The large mass, which was displacing the thecal sac to the right, was removed in several large pieces, tracking down to the L4-5 disc space until all neurologic elements were satisfactorily decompressed. At that time, the annular tear was visualized on the left-hand side; the fragment was sent to pathology and confirmed to be disc material. Confident that the extruded disc material was completely removed, the wound was irrigated and closed uneventfully.

Postoperatively, his leg pain and saddle anesthesia resolved immediately. By the six-week follow-up appointment, he had regained full strength in his ankles and reported no bowel, bladder, or sexual dysfunction.

## Discussion

Lumbar disc herniations are common and typically follow an expected trajectory, most often in a posterolateral direction, and less frequently foraminally or centrally [1]. They will also commonly travel in a rostral or caudal direction. Rarely, the disc fragments can migrate around to the dorsolateral/dorsal side of the thecal sac. This has been described in the literature as a lumbar disc with posterior epidural migration (PEM) or posterior epidural migration of lumbar disc fragments (PEMLDF) [1-2]. As of April 2017, there had been 75 cases reported in the English literature [1], with even fewer occurring in the setting of CES. Additionally, a retrospective case series between February 2007 and June 2010 demonstrated that of 572 patients who underwent surgery for lumbar disc herniation, only six were diagnosed with PEMLDF [3]. This is an important clinical entity to recognize, as up to 55% of these cases are associated with CES [4].

The diagnosis of this rare form of herniated disc is confounded by atypical imaging and symptoms mimicking an abscess, hematoma, facet cyst, or neoplastic process, leading to diagnostic difficulty and uncertainty in management. The reported patient presented a diagnostic challenge due to the relatively nonspecific imaging characteristics seen on magnetic resonance imaging (MRI). Although disc fragments typically are isointense on T1-weighted images and hyperintense on T2-weighted imaging, these findings are nonspecific and can overlap with other diagnoses when present in the dorsal epidural space, such as abscesses, hemorrhage, or synovial cysts [4]. If a contrast study is obtained, PEMLDF tends to display a peripheral enhancement, hypothesized to be due to inflammatory granulation tissue [5]. These enhancement features, though, can cause confusion, as they overlap with other differential diagnoses such as abscesses. However, a report by Zarrabian et al. describes two main features characteristically present (>90%) in PEMLDF: asymmetric soft tissue in the lateral epidural space and soft tissue abutting the intact disc on axial images [4]. Furthermore, discography has been shown to aid in this diagnosis [1]. Our case showed a T2-weighted hyperintense mass with a connection to the dorsal aspect of the L4-5 disc, extending to the left lateral and dorsal epidural spaces, consistent with the aforementioned findings in the literature. We did not obtain a contrasted study nor request a diffusion-weighted imaging (DWI) sequence. A suggestive imaging characteristic for PEMLDF is valuable, given the very different treatment strategies for the differential diagnoses.

Treatment strategies for the components of the differential diagnosis may vary significantly, highlighting the importance of early diagnosis and appropriate clinical suspicion. For example, while a PEMLDF will typically be treated with surgical resection, treatment for an epidural abscess or hematoma may be quite different. Treatment options for an abscess include intravenous antibiotics, percutaneous aspiration, or surgical drainage. Similarly, depending on the neurological status of the patient, an epidural hematoma may require surgery or may be observed. Importantly, however, early diagnosis and treatment of PEMLDF have been shown to correlate with a positive clinical outcome. In a study from 2011, early surgery within three days of symptom onset was one of two main factors contributing to a good or excellent prognosis using the modified Odom criteria [6]. Identifying the correct pathology is, therefore, paramount in providing optimal treatment.

## Conclusions

PEMLDF is rare and provides a diagnostic challenge, sometimes mimicking other, more common posterior epidural etiologies. Characteristics seen on MRI or physical exam can suggest the presence of PEMLDF. This diagnosis, however, requires a high clinical suspicion. Timely diagnosis is of utmost importance, as early surgery has shown to be beneficial for positive clinical outcomes.
